# Noncoding rules of survival: epigenetic regulation of normal and malignant hematopoiesis

**DOI:** 10.3389/fmolb.2023.1273046

**Published:** 2023-10-31

**Authors:** LaShanale Wallace, Esther A. Obeng

**Affiliations:** Department of Oncology, St Jude Children’s Research Hospital, Memphis, TN, United States

**Keywords:** epigenetics, hematopoiesis, histone modification, DNA methylation, clonal hematopoiesis, RNA modification, splicing

## Abstract

Hematopoiesis is an essential process for organismal development and homeostasis. Epigenetic regulation of gene expression is critical for stem cell self-renewal and differentiation in normal hematopoiesis. Increasing evidence shows that disrupting the balance between self-renewal and cell fate decisions can give rise to hematological diseases such as bone marrow failure and leukemia. Consequently, next-generation sequencing studies have identified various aberrations in histone modifications, DNA methylation, RNA splicing, and RNA modifications in hematologic diseases. Favorable outcomes after targeting epigenetic regulators during disease states have further emphasized their importance in hematological malignancy. However, these targeted therapies are only effective in some patients, suggesting that further research is needed to decipher the complexity of epigenetic regulation during hematopoiesis. In this review, an update on the impact of the epigenome on normal hematopoiesis, disease initiation and progression, and current therapeutic advancements will be discussed.

## 1 Introduction

**FIGURE 1:**
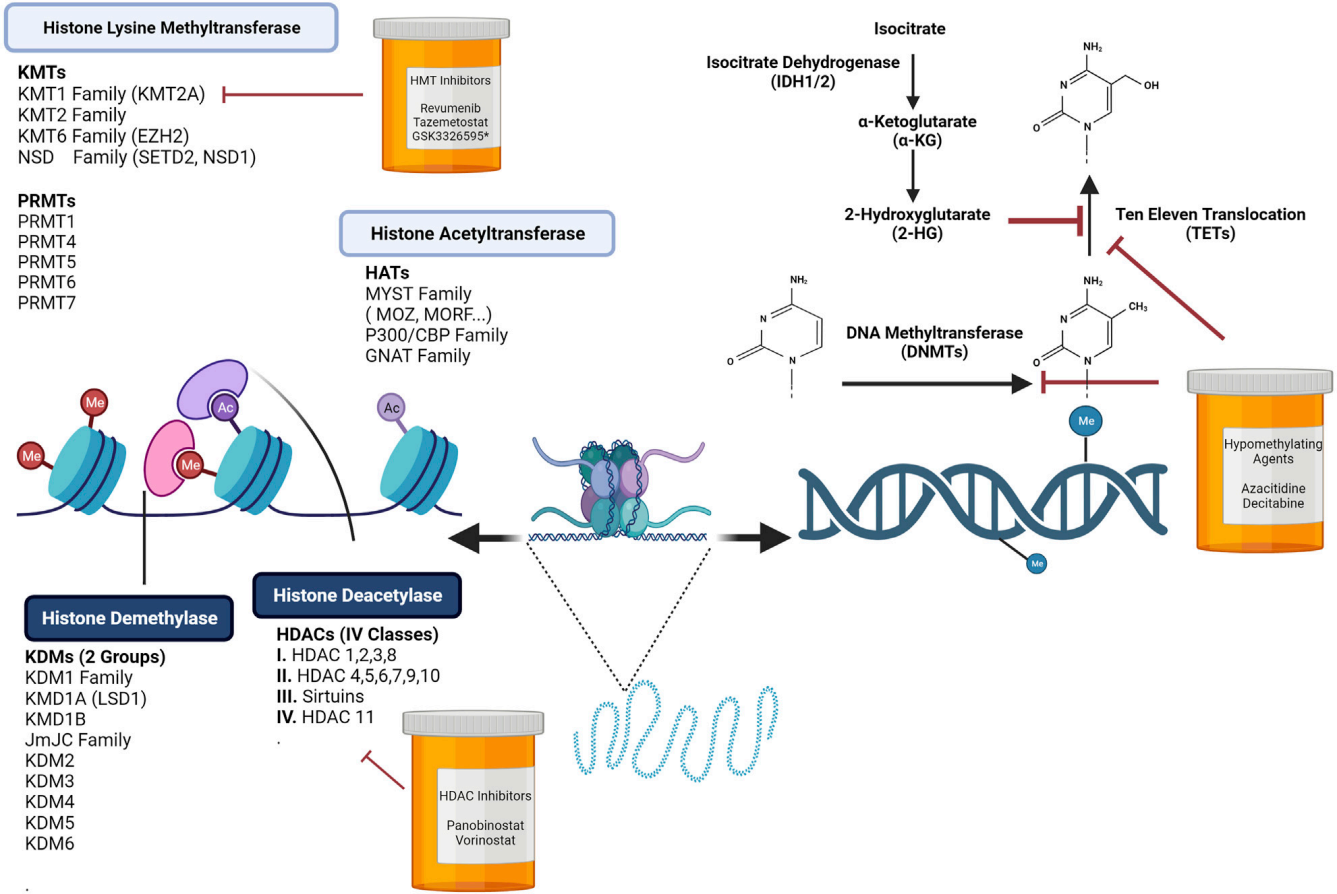
Chromatin Dynamics are Regulated by DNA Methylation Programs and Histone Modifications. Hematopoiesis is controlled by extrinsic and intrinsic regulators. Upon receiving cues from the stem cell niche, epigenetic programs tightly regulate the activation or suppression of self-renewal and differentiation genes. Altering chromatin by covalent histone modifications is one layer of modulation needed to promote or repress transcription. In normal hematopoiesis, epigenetic writers, readers, and erasers play crucial roles in stem cell self-renewal and lineage specification. Disruption or mutations in many of these key regulators can lead to an array of hematological malignancies. Loss or gains in DNA methylation at promoters or enhancers influences the transcription of self-renewal or lineage specific genes. Targeting these key processes has led to the development of histone deacetylase inhibitors, histone acetyltransferase inhibitors, and hypomethylating agents. Figure made with BioRender.

Hematopoiesis is a highly regulated process that sustains the life-long production of blood cells. All mature lineages are derived from a pool of hematopoietic stem cells (HSCs) within the bone marrow. HSCs can self-renew or differentiate, leading to a balance between maintaining an HSC pool throughout life and producing mature cells needed for organism homeostasis. Hematopoiesis was initially thought to occur in a sequential stepwise hierarchy where HSCs contribute equally to each lineage (Cheng et al., 2019). Advancements in lineage tracing and single-cell sequencing experiments have further shed light on the complexity of lineage commitment. In newly proposed models HSCs are still at the top of the hierarchy. However, they are more heterogenous, containing pre-existing lineage biases. Thus, if lineage commitment occurs in a continuum, several extrinsic and intrinsic factors are needed to guide HSCs through the different phases of development. Extrinsically, cues from the niche, including cytokines, growth factors, and nutrients, provide a mechanism to maintain HSC quiescence and prompt differentiation. To complement this network, intrinsic regulators, including transcription factors (TF) and epigenetic regulators, help instruct cell fate decisions. Epigenetics is defined as heritable changes in phenotype or gene expression independent of changes in DNA sequences. Historically, interactions between regulators of DNA methylation and histone modifiers have functioned in a network to remodel the chromatin structure, which in turn determines the gene transcription state (Figure 1). More recently, RNA modifications and their binding proteins have been implicated in playing a homeostatic role in hematopoiesis by regulating isoform expression and transcript stability leading to regulatory feedback of gene expression. Thus, epigenetic regulation balances quiescence, self-renewal, and differentiation in HSCs and, when disrupted, can aid in developing hematological malignancies.

## 2 Role of histone modifications in normal and malignant hematopoiesis

Histone modifications are essential epigenome regulators that control chromatin structure and gene accessibility. Chromatin in an open configuration, termed euchromatin, has less condensed DNA and is readily available for gene expression. In contrast, heterochromatin is more condensed and results in transcriptional repression. Open and closed chromatin states are regulated by directly modifying the chromatin template. Two epigenetic mechanisms that are essential regulators include covalent histone modifications and DNA methylation. Early studies have documented that HSCs have a unique epigenetic footprint creating a highly coordinated progenitor differentiation process by activating and silencing genes leading to the preassembly of transcription factors at lineage-specifying promoters (Weishaupt et al., 2010). There are at least sixteen histone modifications, including but not limited to acetylation, methylation, phosphorylation, ubiquitination, and SUMOylation. Of these histone modifications, methylation, acetylation, and ubiquitination are essential for regulating stem cell self-renewal and differentiation (Weishaupt et al., 2010).

### 2.1 Histone methylation

The establishment of regulatory element reference maps and correlating histone methylation and gene expression patterns in multiple cell types have provided a framework for the identification and activity of promoters and enhancers (Dunham et al., 2012; Yue et al., 2014; Kundaje et al., 2015). Histone methylation primarily occurs at lysine and arginine residues of the core histone protein, H3. Methylation sites associated with the activation of transcription include H3K4, H3K36, and H3K79. In contrast, methylation at H3K9, H3K20, and H3K27 are marks that lead to repressive heterochromatin.

H3K4 methylation is one of the marks of actively transcribed genes (Yang and Ernst, 2017). H3K4 trimethylation (H3K4me3) is typically found over transcription start sites whereas H3K4 dimethylation (H3K4me2) can be enriched at enhancers, transcription start sites, and gene bodies (Orford et al., 2008; Benayoun et al., 2014). Six histone methyltransferases (HMTs) can modify H3K4: MLL1, MLL2, MLL3 MLL4, SETD1A, and SETD1B. Each HMT has a unique function in hematopoiesis. Mll1 expression is important for primitive and definitive hematopoiesis and Hox gene expression (Yu et al., 1995; Hess et al., 1997; Ernst et al., 2004). In adult HSCs, Mll1 regulates the expression of key hematopoietic transcription factor genes including *Hoxa9*, *Pbx1*, and *Mecom* (Jude et al., 2007). Loss of Mll2 expression is associated with inflammatory gene expression defects in macrophages (Austenaa et al., 2012). Hematopoietic specific knockouts of *Mll3* or *Mll4* have shown roles for both of these genes in HSC differentiation into HSPCs, engraftment and protection from oxidative stress (Chen C. et al., 2014; Santos et al., 2014). Setd1a expression is important for erythroid and B-cell differentiation (Tusi et al., 2015; Li et al., 2016) whereas *Setd1b* loss is associated with impaired trilineage maturation and myeloid skewing in conditional knockout mice and self-renewal defects in long term hematopoietic stem cells (LT-HSCs) and lymphoid primed progenitors after transplantation (Schmidt et al., 2018).

HMTs may function as oncogenes or tumor suppressors in leukemogenesis. *MLL1*, located on chromosome 11q23 in humans, is a frequent chromosome translocation partner present in over 70% of infant B-cell acute lymphoblastic leukemia (B-ALL) and 5%–10% of acute myeloid leukemia (AML) (Gu et al., 1992; Tkachuk et al., 1992). MLL translocations are the product of in-frame fusions of the 5′ end of the MLL gene with the 3’ end of one of more than 70 translocation partners, most commonly AF4 and AF9. *MLL1* translocations lead to the overexpression of *HOX* cluster genes and the *HOX* cofactor *MEIS1* (Neff and Armstrong, 2013). MLL3 and MLL4 are tumor suppressors in leukemia. MLL3 is located on chromosome 7q, which is commonly deleted in AML (Chen C. et al., 2014). MLL4 is mutated in 30%–90% of diffuse large B-cell lymphomas (DLBCL) and follicular lymphomas (Morin et al., 2011; Pasqualucci et al., 2011).

Another commonly mutated epigenetic regulator in follicular lymphomas is the H3K27 methyltransferase, enhancer of zeste homolog 2 (EZH2) (Bodor et al., 2013). EZH2 is the catalytic subunit of polycomb repressive complex 2 (PRC2), which negatively regulates transcription via trimethylation of histone three at lysine 27 (H3K27me3). EZH2 and other core components of the PRC2 complex including EZH1, SUZ12, and EED are important for adult stem cell self-renewal and maintenance of pleuripotency (Shen et al., 2008). Overexpression of EZH2 preserves stem cell potential and prevents exhaustion in serial transplantation assays (Kamminga et al., 2006). Deletion of EZH2 in adult murine bone marrow led to comparable wildtype reconstitution of stem cells; however, decreased differentiation of lymphoid cells was observed (Mochizuki-Kashio et al., 2011). Sequencing studies have identified both loss of function and gain of function mutations of EZH2 in various hematologic malignancies suggesting that it acts as both a tumor suppressor and an oncogene. Furthermore, using murine models of AML, Basheer et al. identified diametrically opposed roles of EZH2 during AML induction (tumor suppressor) and maintenance (oncogene) (Basheer et al., 2019). A study examining the impact of inactivating Ezh2 mutations in myeloid malignancies in mice reported that mice developed myelodysplastic disorders that were transplantable and led to the development of myelodysplastic syndrome (MDS), lymphoma, and lymphoproliferative disease (Mochizuki-Kashio et al., 2015). In MDS and T-ALL sequencing studies, EZH2 mutations appear to disrupt the catalytic SET domain resulting in the loss of function of the gene (Ernst et al., 2010; Nikoloski et al., 2010; Zhang et al., 2012). Overexpression of EZH2 has been linked to tumor cell aggressiveness and poor prognosis in multiple myeloma (Pawlyn et al., 2017). Gain-of-function mutations (Y641, A682, and A692) in the catalytic SET domain increases H3K27 methylation resulting in the inhibition of plasma cell differentiation and oncogenesis in B-cell lymphoma.

### 2.2 Bivalent genes

Global analysis of human and murine HSPCs has shed light on the crucial players that help shape multilineage gene priming (Cui et al., 2009; Weishaupt et al., 2010). For instance, studying the features of histone marking led to the identification of the role of bivalent genes in hematopoietic development. Bivalent genes are marked by both active (H3K4me3) and repressive (H3K27me3) histone marks (Azuara et al., 2006; Bernstein et al., 2006). Around 40% of bivalent genes were found to be shared between human and murine HSPCs (Cui et al., 2009; Weishaupt et al., 2010). The most significant number of bivalent promoters were found in HSCs, and a reduction in either the active or repressive mark occurs in genes associated with development and differentiation. Thus, lineage fate decisions are dynamic processes that need histone methylation modifications to maintain the HSPC activation potential required for differentiation (Cui et al., 2009).

### 2.3 Histone demethylation

Histone demethylases erase the existing methylation of histones and are classified into two families: amino oxidase homology lysine demethylase 1 (KDM1), and Jumonji-domain histone demethylase (JHDM). The KDM1 family members KDM1A, also known as lysine specific demethylase 1 (LSD1), and KDM1B demethylate mono- or dimethyllysine. *LSD1* is specific for histone H3 lysine 4 and 9 methylation, which both activate and repress transcriptional programs. In hematopoietic cells, it was shown that *Lsd1* is required for successful differentiation into mature blood cells. A knockout mouse model was utilized to show that conditional inactivation of *Lsd1* led to defects in self-renewal, impairments in HSC differentiation into immature progenitors, and disrupts terminal erythroid and granulocytic maturation (Kerenyi et al., 2013). In erythroleukemia cell lines, LSD1 was shown to mediate the function of key transcription factors TAL1, GATA1, and C/EBPα during erythroid differentiation (Hu et al., 2009; Kohrogi et al., 2021). In many cancers, LSD1 is overexpressed, and studies have demonstrated that LSD1 contributes to the onset and progression of AML.

The second-class of demethylases, JHDM, is composed of 20 members, uses ferrous iron and alpha-ketoglutarate and can demethylate mono-, di-, and trimethyllysine. The JHDM demethylase family is subdivided into KDM2-7 and has yet to be fully studied due to the variation in substrate specificity. Focusing on each subfamily hints that members are essential for normal hematopoiesis and can function as tumor suppressors, oncogenes, or both depending on the cellular context. For example, KDM2b/JHDM1b is important for regulating cell proliferation and senescence, is highly expressed in lymphoid and myeloid leukemias, and is reported to be an oncogene that plays critical roles in both leukemia stem cell self-renewal and leukemogenesis. Depletion of *Kdm2b* led to an impairment of *Hoxa9/Meis1*-induced leukemogenesis, which was partially mediated by decreased expression of p15Ink4b. In contrast, overexpression of *Kdm2b* led to cell growth advantages and suppression of differentiation in normal HSPCs (He et al., 2011). Other studies showed that ectopic expression of KDM2B could antagonize KRAS-driven leukemias, while ablation led to an accelerated KRAS-driven myeloid transformation (Andricovich et al., 2016).

Another demethylase subfamily shown to be required for the growth of MLL-AF9 translocated AML cells is the KDM4 (KDM4A-E) subfamily. Using transplantation assays, it was demonstrated that *Kdm4a*, *Kdm4b*, and *Kdm4c* played functionally redundant roles in hematopoiesis. However, deletion of two or three (Kdm4a, Kdm4b, or Kdm4c) of the enzymes led to a significant reduction in myeloid, T, and B-cell 6 months after transplantation, hinting that KDM4 family members are essential for the maintenance of HSCs (Agger et al., 2019). KDM5 members also have roles in normal and malignant hematopoiesis. KDM5A with NUP98 translocation induced genomic instability in AML, and downregulation led to apoptosis of AML cells (Shokri et al., 2018; Domingo-Reinés et al., 2023). Knockdown of KDM5 was also shown to impact the pathogenicity of leukemia by decreasing leukemic colony forming potential (Xue et al., 2020). Thus, many members of the Jumonji-domain histone demethylase family present as potential therapeutics for various hematological malignancies; however, some members’ roles are context-dependent, making it a bit complex for pharmacological inhibition.

### 2.4 Histone acetylation

Functionally, transcription is enhanced by histone acetylation through the electrostatic interactions between DNA and histones, which in turn cause open active chromatin. Acetylated histones are enriched at promoters and enhancers of active genes and are modulated by two opposing groups of enzymes. Histone lysine acetyltransferases (KAT) acetylate histone proteins by adding an acetyl group to lysine residues resulting in an open chromatin structure accessible for transcription factors. In contrast, histone deacetylases (HDACs) erase acetyl groups from lysines leading to a condensed chromatin structure and gene repression. Opposing roles of KATs and HDACs keep the balance needed to regulate hematopoiesis.

Histone lysine acetyltransferases are a diverse group of proteins that are divided into two classes, Type A and Type B, which are grouped based on being localized within the nucleus or the cytoplasm. Type A KATs function in transcription-related histone acetylation in chromatin and are grouped into five families based on their catalytic domain: P300/CBP, MYST, GNAT, transcriptional coactivators (KAT4 and KAT12), and steroid receptor coactivators (KAT13A-D) (Wapenaar and Dekker, 2016). Type B KATs (KAT1 and HAT4) are mostly cytoplasmic and acetylate newly synthesized histones, which are further transported into the nucleus and integrated into newly synthesized DNA (Wapenaar and Dekker, 2016). Histone acetylation is essential for regulating HSC self-renewal, differentiation, and proliferation in normal hematopoiesis. A large zebrafish screen targeting 425 orthologs of human chromatin factors, led to the identification and validation of 44 factors that affect primitive and definitive hematopoiesis. The 44 identified factors were mapped to a protein network, which displayed that multiple chromatin factor complexes, including those containing the histone acetylases CBP/P300, HBO1, and NuA4, were required for developmental hematopoiesis (Huang H-T. et al., 2013).

CBP/300 and MYST family members have been studied extensively in hematopoiesis, and murine models have shed light on their role in oncogenesis (Wang et al., 2020). Loss of function mutations in *Moz,* a member of the MYST family, lead to a failure of HSC development during embryogenesis. Deletion of *Moz in* adult murine bone marrow cells led to a loss of the long-term repopulating ability of HSCs; however, lineage-committed progenitors were unaffected, suggesting its role in the maintenance of HSCs (Sheikh et al., 2016). A conditional knock-out mouse model of *Mof*, another member of the MYST family, resulted in hematopoietic failure suggesting its critical role in postnatal and adult HSC and progenitor maintenance (Valerio et al., 2017a). MOF orchestrates erythropoiesis through the modulation of chromatin accessibility. Single-cell transcriptomic and bulk chromatin immunoprecipitation sequencing revealed that the expression of MOF is controlled by erythroid specific transcription factors, *Runx1* and *Gfi1b*, through a feed-forward mechanism. Not surprisingly, disrupting MOF expression results in defects in erythroid differentiation, anemia and reduced erythroid progenitors in murine models (Pessoa Rodrigues et al., 2020).

Murine models have demonstrated that CBP and p300 are paralogs with distinct functions in HSC self-renewal and differentiation. *Cpb* heterozygous knockout mice have a decrease in HSCs when aged to 1 year and *Cpb* heterozygous knockout recipients have decreased reconstitution compared to *p300* heterozygous knockout or wild type recipients in secondary bone marrow transplantation assays, suggesting *Cbp* is essential for HSC self-renewal (Rebel et al., 2002). Ablation of *Cbp* in adult conditional knockout mice led to a myeloid bias, lymphopenia, HSC exhaustion, and an increase in quiescent cells demonstrating that *Cbp* is essential for HSC homeostasis (Chan et al., 2011). Specific mutation of the KIX domain in CBP and p300, which binds transcriptional regulators, also have non-redundant functions in hematopoiesis. For instance, hematopoiesis in mice with homozygous mutations in the Cbp KIX domain were essentially normal aside from having slightly lower thymocyte numbers. The same homozygous KIX domain mutations in p300 were associated with megakaryocytic hyperplasia, thymic hypoplasia, anemia, and B-cell deficiency. These changes were also found in homozygous p300 KIX mutant recipients after bone marrow transplantation, suggesting this is a cell intrinsic phenotype (Kasper et al., 2002).

Although these paralogs have distinct roles in normal hematopoiesis, both proteins serve as tumor suppressors. *CBP* loss of function mutations are highly recurrent in patients with DLBCL and follicular lymphoma (Zhang et al., 2017). Studies in transgenic mouse models show that homozygous *Cbp* knockout combined with *Bcl2* overexpression leads to defects in B-cell maturation, Myc overexpression and lymphoma development (García-Ramírez et al., 2017). In AML, The C terminus of CBP is a direct target of chrmosomal translocations to *MLL1* and *MOZ* (Taki et al., 1997; Crowley et al., 2005). *MOF* expression was also found to be required for MLL-AF9 leukemogenesis. Deletion of *Mof* in an Mll-Af9 leukemia mouse model led to reduced tumor burden, decreased colony formation, prolonged survival, and downregulation of genes involved in DNA damage repair. Furthermore, human and murine MLL-AF9 leukemias showed increased sensitivity to a selective small molecule inhibitor of the histone acetyl transferase activity of the MYST protein (Valerio et al., 2017b).

### 2.5 Histone deacetylation

HDACs remove acetylation from histones and promote transcriptional repression. In humans, 18 HDAC enzymes are divided into two families based on the presence of a conserved deacetylase domain and their dependence on specific cofactors. The zinc-dependent HDACs are the deacetylase family, divided into classes I, II, and IV. The class III HDACs require nicotinamide adenine dinucleotide as a cofactor for catalytic function and consist of the sirtuin family proteins (Park and Kim, 2020). HDACs counterbalance acetylation, and any disruption between KAT and HDAC activities can result in aberrant expression of genes and lead to hematologic malignancies.

During hematopoiesis HDACs, participate in various complexes that help modulate the expression of critical genes needed for multilineage development. HDAC1 and HDAC2 are class I HDACs that play an essential role in HSC homeostasis and show compensatory and overlapping functions in hematopoiesis. Conditional knockout of both *Hdac1* and *Hdac2* in the bone marrow leads to bone marrow failure with loss of HSCs and early progenitors due to the dysregulated expression of genes involved in stem cell survival and maintenance. *Hdac1* or *Hdac2* single knockout is associated with decreased B-cell numbers but preserved myeloid differentiation. Only *Hdac1* knockout was associated with decreased erythroid colony formation and a defect in the expansion of early erythroblasts (Marinus et al., 2014). The expression of *HDAC1* changes throughout lineage specification, making it subject to regulation by transcription factors such as GATA2, C/EBP, and GAT1. During differentiation of common myeloid progenitors (CMP), GATA2 and C/EBP represses HDAC1, while GATA1 activates it during erythroid megakaryocytic differentiation (Wada et al., 2009). *Hdac8*, another class 1 member, was shown to be an essential regulator of long term hematopoietic stem cell (LT-HSC) function by maintaining long-term hematopoietic repopulation and protection from stress (Hua et al., 2017).

Other classes of HDACs are essential in HSC homeostasis and aging. Inhibition of the class II HDAC, *HDAC5*, leads to increased acetylated p65 in the nucleus of HSCs which increases expression of CXCR4 and enhances HSC homing (Huang et al., 2018). Members of the sirtuin family regulate HSC aging. For example, *Sirt1* deletion in young murine HCSs led to an accumulation of DNA damage and features of aged HSCs including myeloid skewing and anemia (Rimmelé et al., 2014). *Sirt3* is highly enriched in HSCs, where it functions to reduce stress. The role of *Sirt3* in HSC maintenance is dispensable in young mice but essential under stress or in aged mice (Brown et al., 2013). Lastly, inactivating *Sirt7* decreased quiescence, disrupted the regenerative capacity of HSCs, and increased mitochondrial protein folding stress. Upregulation of *Sirt7* improved regenerative capacity in aged HSCs, further hinting that HDACs are highly dynamic in normal hematopoiesis (Mohrin et al., 2015).

*HDAC1* and *HDAC2* are upregulated in DLBCL, peripheral T-cell leukemias, cutaneous T-cell lymphomas and NK/T-cell lymphomas and the expression of HDAC1 was found to be related to worse prognosis in patients (Min et al., 2012). In B-cell lymphoma HDAC1 and HDAC2 inhibition repressed proliferation suggesting an oncogenic role in lymphoma. In contrast, HDAC4 expression is low in widespread hematological malignancies suggesting a protective tumor suppressor role (Wang et al., 2020). HDACs are also critical for the oncogenic potential of leukemia fusions through the recruitment and repression of genes responsible for hematopoietic differentiation. Thus, HDACs are great targets for clinical drug development.

### 2.6 Histone ubiquitination

Polycomb repressive complex 1 (PRC1) catalyzes the monoubiquitination of lysine 119 on H2A, can condense nucleosomes, and exists in multiple canonical (PRC.1 and 1.4) and non-canonical variants (PRC1.1, 3, 5, and 6) (Zepeda-Martinez et al., 2020; Nakajima-Takagi et al., 2023). The different forms of PRC1 are divided according to the subtype of the Polycomb group ring finger (PCGF) subunits (PCGF1-6) (Nakajima-Takagi et al., 2023). Members of the canonical PRC1.4, such as BMI1, are highly expressed in HSCs and have been linked to lymphocyte development and malignant transformation (Park et al., 2003). To understand the molecular mechanisms by which PRC1 variants contribute to the self-renewal of HSCs, Park et al. analyzed gene expression profiles of highly purified LT-HSCs and found that *Bmi1* was expressed. Additionally, using competitive repopulating assays, researchers showed that *Bmi1* null mice had a decreased capacity to reconstitute mature lineages and lost most of the donor-derived HSCs by week 10, suggesting that *Bmi1* is required for self-renewal of adult HSCs (Park et al., 2003). In addition, forced expression of *Bmi1* enhanced symmetrical cell divisions of HSCs and led to the *ex vivo* expansion of multipotent progenitors (Iwama et al., 2004). Multiple studies have shown that BMI1 regulates self-renewal and maintains multipotency by transcriptionally repressing *CDKN2*, *p16Ink4*, and *p19Arf* (Park et al., 2003; Nakajima-Takagi et al., 2023). Studies also show that *Bmi1* knockout mice have a block in B-cell differentiation by silencing the promoter of *Ebf1* and *Pax5*, B-cell-specific transcription factors (Oguro et al., 2010).

Other members of both the canonical and non-canonical PRC1 were also shown to regulate HSCs. *Mel18* was shown to promote self-renewal and increase quiescence acting as a negative regulator of differentiation (Kajiume et al., 2004). The PRC1 complex is highly dynamic, with the chromobox (CBX) family proteins either leading to increased self-renewal and induction of leukemia (*Cbx7*) or differentiation and exhaustion of HSCs (*Cbx2,4,8*) when overexpressed (Klauke et al., 2013). Noncanonical PRC1 complex member *BCOR* is ubiquitously expressed across adult tissues, and mutations within this gene occur in acute myeloid leukemia (AML), myelodysplastic syndrome (MDS), chronic myelomonocytic leukemia (CMML), and aplastic anemia (Kelly et al., 2019). Non-canonical PRC1 components including polycomb group ring finger protein 1 (*Pcgf1*), *USP7* and *TRIM27* appear to have tumor suppressor functions in myelofibrosis and AML (Maat et al., 2021; Shinoda et al., 2022).

## 3 DNA methylation: A key modification for development and differentiation

DNA methylation is a key epigenetic modification for development, stem cell differentiation, and oncogenesis (Celik et al., 2015). Methylation is associated with an inhibitory role near transcriptional start sites and active transcription in gene bodies. In mammalian cells, DNA methylation is defined as the covalent transfer of a methyl group from S-adenyl methionine to the 5’ position of the cytosine ring of DNA by a family of DNA methyltransferases (DNMTs). The majority (98%) of DNA methylation occurs within CpG dinucleotides in somatic cells. In contrast, around a quarter of methylation occurs in a non-CpG context in embryonic cells (Lister et al., 2009). However, this non-CpG methylation is not observed as cells differentiate and mature.

### 3.1 DNA methylation

DNA methylation is regulated by three main DNMTs that help maintain and establish DNA methylation. DNMT1, the first identified DNMT gene, is a canonical maintenance methyltransferase that targets hemi-methylated DNA sequences and is critical in re-establishing the methylation landscape after DNA replication (Haggerty et al., 2021). DNMT3A and DNMT3B are responsible for establishing methylation signatures in different cell types and also transfer a methyl group to unmethylated DNA during development. Structurally, all major DNMTs contain a C-terminal catalytic domain, a central linker, and a regulatory motif within the N-terminal region. DNMT3A and DNMT3B have a PWWP, ATRX, and PHD domain. However, DNMT1 is unique, containing distinct domains such as PBD, DRFTS, and BAH (Brunetti et al., 2017). Over the years, research has provided perspective on the significant roles DNMTs have on hematopoiesis. Hematopoietic stem cells are tasked with replenishing stem cell pools through self-renewal and producing mature cell lineages through differentiation. Keeping the equilibrium between both processes is extremely important and is controlled by extrinsic and intrinsic signals. Intrinsically, DNA and histone modifications help define the gene expression patterns needed as stem cells decide to self-renew or differentiate from HSCs to progenitors, which go on to differentiate into mature lymphoid, erythroid, and myeloid cells.

Studies of the individual DNMTs have linked their function to self-renewal and lineage-specific fate decisions. Through transplantation studies, it was shown that in addition to maintaining parental cell methylation patterns, *Dnmt1* also possesses a critical role in cell state transitions of adult stem cells (Trowbridge et al., 2009). In particular, reducing *Dnmt1* in mouse HSCs led to impaired self-renewal and multilineage differentiation (Trowbridge et al., 2009). Gene expression and methylation analysis highlighted that Dnmt1 deletion causes a distinct pattern in LT-HSCs, short-term hematopoietic stem cells (ST-HSCs), multipotent progenitors (MPPs), and myeloid progenitors. Further integration of mouse HSPCs highlighted that there is a bias toward the myeloid erythroid lineage through DNA hypomethylation, which caused the increased expression of transcription factors *Gata1*, *Id2*, and *Cepba*, but a decrease in lymphoid and stemness genes (Bröske et al., 2009).

In contrast, DNMT3A and DNMT3B are *de novo* methyltransferases linked to transcriptionally silencing self-renewal genes in HSCs. Knockout models of *Dnmt3a* led to the finding that self-renewal was favored over differentiation in mouse HSPCs. Deletion led to hypo and hypermethylation of specific loci, resulting in the upregulation of self-renewal genes such as *Runx1* and *Gata3*. Compared to wildtype cells, *Dnmt3a* deficient cells can extensively expand the HSC pool; however lineage output does not increase in a similar proportion suggesting that there is a differentiation defect induced by loss of *Dnmt3a*. The combined loss of *DNMT3A* and *DNMT3B* is synergistic, leading to a severe block in differentiation and enhanced self-renewal through β-catenin activation. *DNMT3B* deficiency has minimal effects on HSCs hinting that *DNMT3A* can compensate for *DNMT3B* loss, further highlighting that *DNMT3A* is essential for HSC differentiation. DNA methylation is a dynamic process, and understanding the role of DNA methylation in cellular lineage commitment is still being elucidated. Evidence shows that cellular identity is defined by epigenetic switches that may act as gatekeepers that prevent differentiated cells from aberrantly expressing stem cell-associated genes (Bock et al., 2012).

### 3.2 DNA demethylation

DNA demethylation also plays a considerable role in regulating HSC differentiation. Studies show various instances where lineage-specific demethylation is required for the upregulation of critical genes such as *Gadd45a*, an essential gene implicated in myeloid development during the common myeloid progenitor (CMP) to granulocyte-monocyte progenitor (GMP) transition (Cullen et al., 2014). The demethylation process is complex and is orchestrated through a protein family known as the ten-eleven translocation (TET) protein. The TET proteins belong to a family of dioxygenase enzymes subdivided into TET1, 2, and 3. All three TET proteins have similar catalytic activity by functioning to oxidize the methyl group on 5mC, yielding 5- hydroxymethylcytosine, 5- formylcytosine, and 5- carboxylcytosine (Bowman and Levine, 2017). The *TET1* and *TET3* genes encode for both the DNA binding domain, the CXXC domain, and the catalytic domain. Due to chromosomal inversion during vertebrate evolution *TET2* was separated from its DNA biding region, which is encoded by a neighboring gene, IDAX. The deposition of 5hmC on CpG islands vs gene bodies by TET proteins is thought to be influenced by the type of DNA binding domain (CXXC or IDAX) (Huang et al., 2014). TET1 is usually detected at promoters or enhancers in mouse embryonic stem cells, while TET2 is enriched in enhancer regions and gene bodies. TET2 and TET3 are ubiquitously expressed in all HSPC compartments, with TET2 having decreased expression in megakaryocyte-erythroid progenitors (MEPs) and TET3 having reduced levels in differentiated populations. Assessment of the TET proteins in normal hematopoiesis using knock-down studies have suggested that TET2 is responsible for 60% of DNA dioxygenase activity in HSPCs highlighting its essential role in HSPCs (Guan et al., 2021). Reduction of Tet2 in murine HSPCs results in a competitive advantage over wild-type cells and an increase in colony formation ability after serial replating, implicating a role in HSC self-renewal (Moran-Crusio et al., 2011; Rasmussen et al., 2015a). Gene expression profiles of serial replated cells had an increase in expression of self-renewal factors *Meis1* and *Evi1*. Increasing evidence has suggested that TET proteins act on regions outside of CpG islands, and 5hmc analysis shows that it is enriched on enhancer elements and CTCF insulators. Thus, loss of *Tet2* in HSCs led to genome-wide hypermethylation of enhancers and changes in gene expression of both tumor suppressors and oncogenes (Hon et al., 2014; Rasmussen et al., 2015a; Rasmussen et al., 2015b). *Tet2* null mice develop many hematopoietic abnormalities with myeloid proliferative neoplasms and chronic lymphocytic leukemia, hinting that *Tet2* is also a tumor suppressor in hematopoietic tissue.

## 4 DNA methylation in disease and therapeutic implications

**FIGURE 2:**
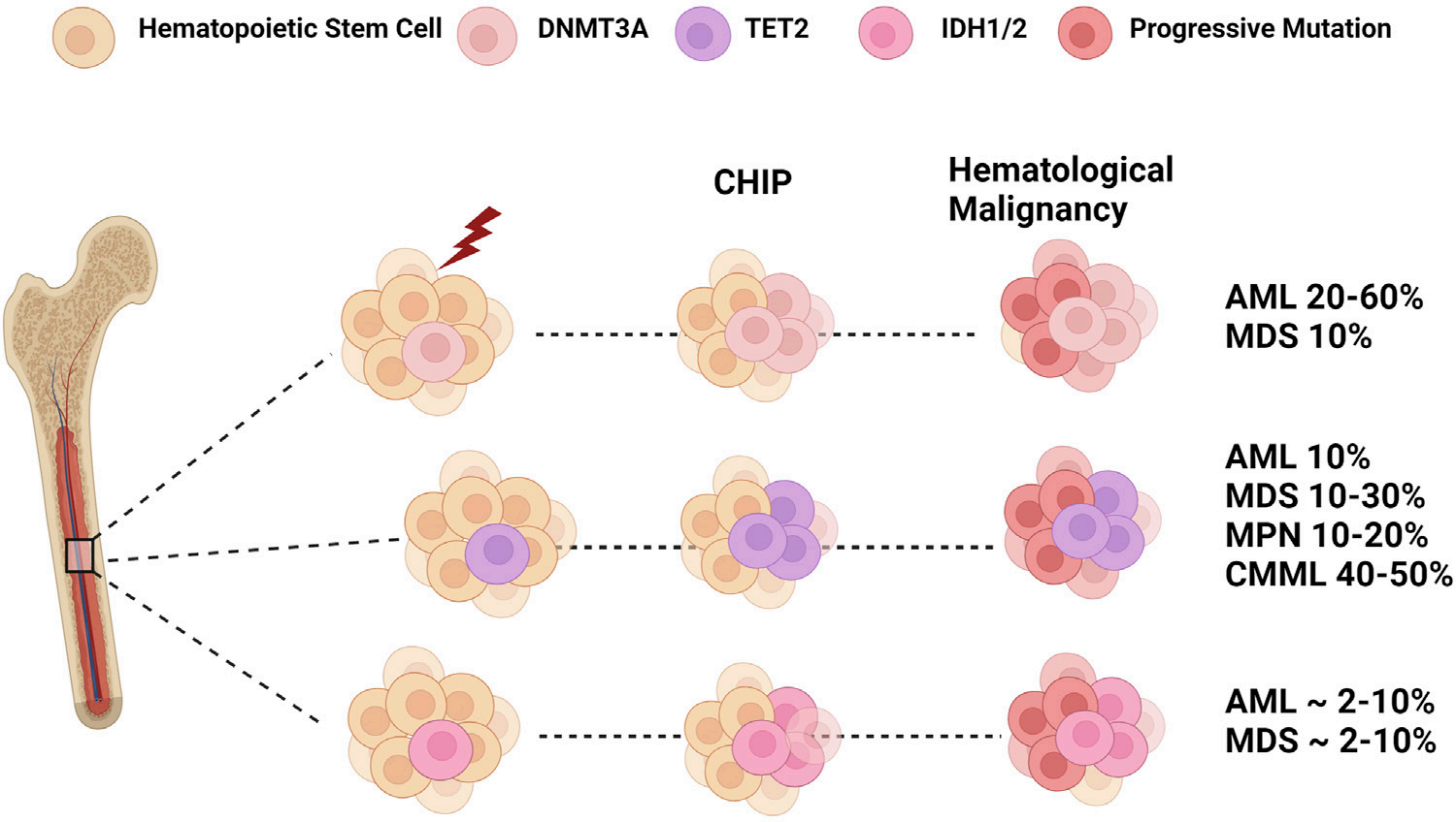
Epigenetic Dysregulation: From CHIP to Leukemia. Clonal hematopoiesis is a disease caused by the clonal expansion of stem clones harboring leukemogenic mutations. Mutations in *DNMT3A* and *TET2* are two of the most frequent genes found in CHIP. In addition, IDH mutations are also associated with CHIP in older populations. Selective pressures such as age and environmental insults lead to the expansion of these mutant clones that may progress to myeloid malignancies such as myelodysplastic syndrome (MDS), myeloproliferative neoplasms (MPN), and chronic myelomonocytic leukemia (CMML). The subsequent acquisition of additional mutations in genes such as FLT3 and NPM1 can result in the transformation into overt acute myeloid leukemia (AML). Figure made with BioRender.

Disrupting the balance between stem cell self-renewal and lineage commitment can lead to the onset of hematological malignancies. Genomic sequencing studies have revolutionized the understanding of disease pathogenesis by revealing that malignancy arises from acquired somatic mutations in genes involved in specific cellular pathways, including epigenetic regulation, chromatin modification, and RNA splicing. However, the mechanisms by which these mutations lead to normal HSCs transforming into leukemic stem cells remain poorly understood. More recently, studies in healthy individuals revealed that somatic mutations in HSCs leading to clonal expansion are acquired during aging. Clonal hematopoiesis of indeterminate potential (CHIP) is the expansion of a hematopoietic stem cell clone with an acquired driver mutation in individuals without cytopenias or overt malignant hematologic disease (Genovese et al., 2014; Jaiswal et al., 2014). CHIP studies have identified a high prevalence of gene mutations that overlap with those found in overt hematological malignancies such as MDS, AML, and chronic myeloid leukemia (CML). CHIP represents a pre-disease state or the first step in the path of leukemogenesis. As mutant CHIP clones expand, the acquisition of additional driver mutations can lead to MDS and further transformation into AML. The most frequently mutated genes in CHIP are the epigenetic regulators *DNMT3A*, *TET2*, and *ASXL1*. IDH1 and IDH2 are metabolic enzymes that can affect TET2 function when mutated in CHIP or AML (Figure 2). Thus, epigenetic regulators remain attractive targets for myeloid and lymphoid malignancies. DNA hypomethylating agents (HMAs) 5-azacytidine and decitabine are approved treatments for MDS and AML. Azacitidine and decitabine are cytidine analogs that cause DNA demethylation by inactivation of DNMT-1. However, some patients develop primary and secondary treatment failures, showing that the “one-size fits all” approach needs adapting (Prébet et al., 2011; Nair et al., 2021).

Mutations in *DNMT3A* are found in ∼10% of MDS and ∼30% of AML patients. In all instances, *DNMT3A* occurs early in transformation, making it an attractive target for new therapeutic approaches. *DNMT3A* mutations in CHIP and AML are primarily missense, with residue R882 being the most affected. In particular, the R882H variant leads to a dominant-negative effect over the wildtype protein, resulting in altered methyltransferase activity and genome-wide hypomethylation in patients (Russler-Germain et al., 2014; Langstein et al., 2018). Murine models have been instrumental in understating the link between *DNMT3A* and leukemogenesis. In particular, serial transplantation assays of *Dnmt3a* null cells did not lead to overt malignancy (Challen et al., 2012), suggesting that transformation into leukemia requires sequential mutations. Consequently, a long-term survival study of *Dnmt3a* null HSCs decreased overall survival compared to WT and developed a spectrum of myeloid and lymphoid malignancies. The variations in disease further support that DNMT3A confers a preleukemic phenotype that acquires additional mutations during transformation (Mayle et al., 2015).

In a large cohort of *de novo* AML patients, *DNMT3A* mutations commonly co-occurred with *NPM1*, *FLT3-ITD*, *IDH1/2*, and *FLT3-TKD* mutations (Torabi et al., 2022). Consequently, modeling *Dnmt3a* commutations in murine models has provided additional mechanistic insight. Conditional *Dnmt3a* R878H (equivalent R882 mutation) and *Nras* G12D double knock-in mice developed aggressive AML, mediated by an upregulation in *Myc* and its target genes (Shi et al., 2019). Similar studies have also demonstrated that *Dnmt3a* deletion or mutations cooperate with Flt3ITD (Poitras et al., 2016), *Npm1c* (Guryanova et al., 2016), or *cKIT* (Celik et al., 2015) in leukemia transformation. Interestingly, a model of Dnmt3a-driven CHIP demonstrated that acquiring *Npm1* mutations can lead to the development of myeloproliferative disorders (MPD), and additional transplantation leads to AML (SanMiguel et al., 2022).

Somatic mutations in *TET2* are frequently identified in individuals with CHIP and various lymphoid and myeloid hematological malignancies. *TET2* mutations occur throughout the gene and can include missense mutations found in its functional domain affecting the catalytic activity of TET2. Loss of function mutations in *TET2* have been reported in 20% of MDS and 50% of patients with chronic myelomonocytic leukemia (CMML). Human and murine studies have shown that *TET2* mutations occur in HSCs and are early events that skew differentiation toward the myeloid lineage (Delhommeau et al., 2009; Ko et al., 2010). For instance, using a competitive transplantation assay Moran-Crusio et al. validated that Tet2 regulated HSC self-renewal by showing that Tet2 knockout HSPCs out compete wild type cells and expand HSCs and GMPs (Moran-Crusio et al., 2011). It was also shown in different models that *Tet2* loss led to the development of features of human CMML, which was characterized by myeloproliferation and splenomegaly (Li et al., 2011; Moran-Crusio et al., 2011; Quivoron et al., 2011). Xenotransplantation studies of *TET2* mutant MPN samples that demonstrated mutant cells could engraft NOD-SCID mice (Delhommeau et al., 2009). Patient cohorts show that *TET2* mutations are associated with low 5hmC levels in the bone marrow compared to healthy controls (Ko et al., 2010). Likewise, it was also demonstrated that deletion of *Tet2* led to a reduction in 5hmC with a concomitant increase in the 5mC levels in the DNA of BM cells (Hon et al., 2014).

TET2 can also influence the lymphoid lineage, and multiple mutations have been reported in angioimmunoblastic T-cell lymphomas, DLBCL, and mantle cell lymphoma (Quivoron et al., 2011; Meissner et al., 2013; Dominguez et al., 2018). Interestingly, a study assessing the catalytic dependent and independent requirements for Tet2 found that mice with catalytically inactive mutant mice developed myeloid malignancies reminiscent of MDS (Ito et al., 2019a). In contrast, *Tet2* knockout mice developed both myeloid and lymphoid malignancies. Gene expression profiles differed between the two models, and Tet2-knockout HSPCs had decreased levels of lineage specifiers *Gata2* and *Hoxa9*. These findings suggest that *TET2* mutations lead to different hematological malignancies that may depend on the catalytic activity of TET2 (Ito et al., 2019a).

Like *DNMT3A*, *TET2* is considered an early preleukemic mutation associated with the sequential acquisition of additional mutations in AML and myeloproliferative diseases. In AML, *TET2* mutations are commonly commutated with secondary driver genes such as *FLT3* and *JAK2*-V617F (Rasmussen et al., 2015a). Multiple mouse models have been developed to understand the underlying mechanisms both mutations have together in hematopoietic neoplasms. When *Tet2* deficient mice were crossed with Flt3ITD mice, hypermethylation of specific genomic loci, such as the *Gata2* promoter, was associated with reduced expression and the development of transplantable leukemia (Shih Alan et al., 2015). In an MPN model, mice with *Jak2*V617F and *Tet2* mutations had a more severe MPN phenotype, where double mutant HSPCs could maintain MPNs in secondary recipients after transplantation (Chen et al., 2015). In this context, *Tet2* was speculated to have different roles depending on the stage of the disease. In already established MPNs, *TET2* loss may accelerate malignancy, whereas *TET2* as the initial mutation may serve a role in initiating MPNs, as shown in CHIP (Kameda et al., 2015). Thus, *TET2* mutations are context dependent, and additional studies to determine how different contexts of mutation acquisition lead to paradoxical changes in disease progression are still warranted.

In many cases, *DNMT3A* and *TET2* are commutated. Having mutations of two driver genes has ushered the field to investigate how these genes with opposite functions synergize during disease. In a conditional knockout model of *Tet2* and *Dnmt3a*, mice developed accelerated malignancy by leading to the upregulation of lineage specific TFs and obstructing differentiation (Zhang et al., 2016).

Perturbations in the expression of the two other TET enzymes are less frequent in hematologic malignancies. In rare cases of AML, *TET1* is fused to *MLL*. A large genome wide expression study of 100 AML patient samples and nine normal bone marrow samples revealed that *TET1* is over-expressed in M*LL*-rearranged AML (Huang H. et al., 2013). Additionally, chromatin immunoprecipitation assays revealed that *TET1* was a direct target of *MLL* which binds directly to the promoter of *TET1* (Huang H. et al., 2013). Silencing of *Tet1* led to downregulation of MLL target genes including *HOXA9*, *PBX3*, and *MEIS1*. In this context, *TET1* is shown to act as an oncogene instead of a tumor suppressor like *TET2* and *TET3*. Rare *TET1* and *TET3* mutations have been found in other hematological malignancies including AML (*TET1*), T-cell lymphoma (*TET3*) and chronic lymphocytic leukemia (*TET1* and *TET3*) (Langstein et al., 2018).

Genomic sequencing studies revealed that isocitrate dehydrogenase 1 and 2 (*IDH1* and *IDH2*) were a new class of genes mutated in myeloid malignancies (Losman et al., 2013). The main function of these homodimeric metabolic enzymes is to convert isocitrate to α-ketoglutarate (α-KG) while producing reduced NAPDH from NADP^+^ and CO_2_. IDH1 (located in the cytoplasm and peroxisomes) and IDH2 (found in the mitochondrial matrix) are highly homologous to each other (Cairns and Mak, 2013). *IDH1* and *IDH2* missense mutations occur around 8% and 12% in AML, respectively. The most common mutation in *IDH1* is R132, and the most common mutations in *IDH2* are R140 and R172 (Cairns and Mak, 2013). These common mutations result in a neomorphic enzymatic activity, which catalyzes NADPH and α-KG to produce active metabolite D 2-hydroxygluterate (D-2HG). The two main targets of D-2HG are the KDM family of HDACs and the TET family of enzymes, and due to its structural affinity with α-KG, D-2HG competitively inhibits both families of enzymes. Inhibition with 2-HG leads to increased H3 lysine methylation and global hypermethylation. IDH1/2 and *TET2* mutations are mutually exclusive. *TET2* knockout and *IDH2* mutations have similar effects on hematopoietic differentiation, suggesting that the effects may be due to *TET2* inhibition by D-2HG (Figueroa et al., 2010). IDH genes are also considered preleukemic genes and are detected at very low levels in CHIP compared to other epigenetic regulators. The role of IDH1 and IDH2 in normal hematopoiesis is still being determined. However, several studies have provided insights into functions other than regulation of TET enzymes. For instance, *Idh1* mutations in a murine model increased H3K9 methylation, leading to the downregulation of the DNA damage sensor *Atm*. Decreased Atm impaired DNA repair and reduced HSC self-renewal. ATM was also found to be decreased in human AML with *IDH1* mutations suggesting that IDH1 can alter cellular differentiation by changing the histone code (Inoue et al., 2016). A study performed in human cell lines found that the R132H mutation in IDH1 could block differentiation and promote cytokine independence (Losman et al., 2013).

Over the past several years clinical trials of IDH protein inhibition have demonstrated promising response rates in relapsed-refractory AML and there are currently several small molecule inhibitors that have been approved by the FDA for the treatment of *IDH1* (ivosidenib, olutasidenib) or *IDH2* (enasidenib) mutant AML (Gruber et al., 2022). For instance, a phase 1 trial of enasidenib, a selective small-molecule inhibitor of IDH2 resulted in an overall response rate of 40.3% (Stein et al., 2017). In addition, a phase 1 clinical trial for IDH1 inhibitor ivosidenib resulted in 41.6% overall survival (DiNardo et al., 2018; Zhuang et al., 2022). As a single agent, ivosidenib led to 30.4% complete morphological remission in patients with mutant IDH1, however many of the patients eventually acquired drug resistance or relapsed.

A study aiming to elucidate mechanisms that dictate response to ivosidenib, developed a novel inducible Idh1 mouse model with co-expressed *Dnmt3a* and *Nras* mutations (Gruber et al., 2022). Mice expressing all three mutations developed AML and had improved survival after treatment with ivosidenib when compared to the vehicle treated mice. Throughout treatment mutant cells were still detected, highlighting that sole IDH inhibition does not fully inhibit the disease. Further analysis revealed that ivosidenib promoted cycling of leukemia stem cells and increased the expression of pyrimidine salvage components, which led the authors to test a combination treatment of ivosidenib and azacitidine. The study demonstrated that ivosidenib leads to the uptake of azacitidine in immature leukemic cells, further causing changes in DNA methylation at promoters and upregulation of genes that promote myeloid differentiation (Gruber et al., 2022).

Mutations in metabolic enzymes such as IDH1 and IDH2 are an example of the dynamic role metabolism has in hematopoiesis. Metabolic enzymes not only modulate epigenetic regulators but are also hijacked by cancer cells to promote disease progression and drug resistance (Patel et al., 2022). The adaptation to energy requirements from glycolysis to oxidative phosphorylation as HSCs differentiate has become a hot topic in the field as highlighted in several recent reviews exploring the metabolic changes that occur during HSC development, stress, and leukemic transformation (Ito et al., 2019b; Nakamura-Ishizu et al., 2020; Patel et al., 2022).

## 5 RNA modifications in normal and malignant hematopoiesis

Improvements in RNA sequencing technologies and stem cell specific disease models have established connections between RNA epigenetics and chromatin structure, divulging the roles of RNA binding proteins (RBPs) and RNA modifiers in the epigenetic regulation of HSC genome function.

### 5.1 RNA splicing

Messenger RNA (mRNA) splicing is the dynamic process whereby noncoding (intron) or coding (exon) nucleotide sequences are removed from a premature mRNA transcript to generate a mature transcript or isoform that will subsequently be translated into protein. Alternative mRNA splicing is a co-transcriptional process that increases the functional diversity of proteins through the generation of tissue-specific isoforms (Wang et al., 2008). Alternative splicing can also lead to decreased protein expression by causing the inclusion of introns or exons containing a premature termination codon (PTC). The presence of a PTC can lead to induction of the nonsense mediated mRNA decay (NMD) pathway, a quality control pathway that degrades these aberrantly spliced transcripts (Belgrader et al., 1994). Bulk RNAseq studies performed in primitive HSPC populations including human fetal liver, cord blood, and bone marrow CD34^+^ cells revealed differences in the isoform expression of transcription factors including *HMGA2 and MEIS1*, and the epigenetic regulator, *DNMT1*. However, these developmentally distinct CD34^+^ cell populations had little to no difference in the expression of these genes, highlighting the importance of alternative splicing in regulating stage-specific features of HSPCs during development (Cesana et al., 2018).

RNAseq studies performed in human CD34^+^ HSPCs and downstream myeloid and lymphoid progenitor populations have documented the presence of lineage-specific alternative splicing events associated with differential isoform expression during myeloid and lymphoid lineage commitment (Liu et al., 2011; Chen L. et al., 2014). Pathway analyses of the alternatively spliced genes found them to be enriched in pathways important for hematopoietic differentiation and cell cycle progression such as the Wnt/β -catenin and Rac/RhoA signaling pathways. A highly regulated alternative splicing program was identified in human erythroblasts differentiated *in vitro* to progress through the last four cell divisions before enucleation (Pimentel et al., 2014). This alternative splicing program causes tissue-specific isoform expression of genes important for chromatin condensation, autophagy and enucleation as well as NMD-associated splicing events that are thought to decrease the expression of RNA and DNA binding proteins and histone modifying enzymes that are presumably not needed in late-stage erythroblasts. Orchestrated intron retention programs have been shown to be important to regulate the expression of splicing factors, among other genes, during terminal erythropoiesis (Edwards et al., 2016; Pimentel et al., 2016), granulocyte maturation (Wong et al., 2013), and B-cell development (Ullrich and Guigo, 2020) through both nuclear retention and NMD of the alternatively spliced transcripts.

Given the importance of mRNA splicing to HSC function and hematopoietic differentiation, it is not surprising that mutations in components of the mRNA splicing machinery occur early in leukemogenesis and are among the most frequently identified somatic lesions in cancer (Graubert et al., 2011; Papaemmanuil et al., 2011; Yoshida et al., 2011). The most commonly mutated spliceosome genes in myeloid malignancies, *SF3B1*, *SRSF2*, and *U2AF1,* are also frequently identified in individuals with CHIP (Genovese et al., 2014; Jaiswal et al., 2014). Interestingly, while patient-associated point mutations in each of these genes have been shown to lead to an expansion of HSCs and HSCPs that is associated with cytopenias and dysplastic cellular features in murine models, these mutations also have detrimental effects on HSC self-renewal in competitive transplantation assays (Kim et al., 2015; Shirai et al., 2015; Obeng et al., 2016).

Mutations in *SF3B1*, *SRSF2*, and *U2AF1* are uniformly heterozygous and mutually exclusive (Yoshida et al., 2011), findings which have led to the hypothesis that the remaining wild-type allele is essential for cells with splicing factor mutations and fueled efforts to target spliceosome function in patients with splicing factor-mutant myeloid malignancies (Lee et al., 2016; Fong et al., 2019). Clinical trial results to date are mixed, with on target effects noted but little effect on disease progression (Steensma et al., 2019). This may be due to an incomplete understanding of which aberrant splicing events are essential for myeloid leukemogenesis. Recent preclinical studies that may hold promise for selectively targeting splicing factor-mutant cells include the development of “synthetic intron” containing constructs which are only properly spliced to express toxic proteins in cells that contain SF3B1 mutations (North et al., 2022) and findings that SF3B1 mutations may confer sensitivity to replication stress induced by PARP inhibition (Bland et al., 2023).

### 5.2 Non-coding RNAs

RNA regulatory networks play vital roles in hematopoiesis and work in concert with histone modification and DNA methylation programs to regulate gene expression. Post-transcriptional modifications extensively studied in normal and disease states include microRNAs (miRNAs) and long-non-coding RNAs (lncRNAs). miRNAs are small non-coding RNAs consisting of 19-24 nucleotides that regulate gene expression by binding to mature mRNAs and causing post-transcriptional silencing of their targets. Since the discovery of the first miRNA in 1993, recent reports estimate that the human genome encodes approximately 2,600 mature miRNAs (Plotnikova et al., 2019).

In normal hematopoiesis miRNAs help regulate stem cell self-renewal, differentiation, cell cycle and lineage specification. MiR-126, miR-125a/b and miR-129a regulate self-renewal of hematopoietic stem cells by repressing multiple targets (Lechman Eric et al., 2016). miR-126 is highly expressed in murine and human HSC compartments, and plays a vital role in maintaining HSC quiescence by restricting cell cycle progression (Lechman Eric et al., 2012). Researchers found that decreasing miR-126 expression in cord blood HSPCs resulted in increased proliferation of HSPCs.

During lineage specification microRNAs are implicated in erythropoiesis, megakaryopoiesis, granulopoiesis, and lymphopoiesis. During primitive hematopoiesis miR-126 expression helps regulate erythroid differentiation by increasing erythroid progenitors through Vcam-1 (Sturgeon Christopher et al., 2012). Studies found that miR-144 and miR-451 are direct targets of *GATA-1* and are required for erythropoiesis (Dore et al., 2008), while miR-223 plays a vital role in granulopoiesis through a negative feedback loop involving *NF1-A* and *C/EBPα* (Fazi et al., 2005). Additionally, miR-126 specific interaction with *c-myb* was shown to control thrombocyte-erythrocyte lineage decisions (Grabher et al., 2011). In lymphopoiesis, miR-150 was upregulated during the development of T and B cells and when overexpressed in HSPCs the transition from the pro-B to the pre-B cell stage is blocked (Zhou et al., 2007).

Given the role of miRNAs in regulating quiescence and differentiation, it is not surprising that alterations in miRNA expression can lead to the development of hematological malignancies in which miRNAs can act as either tumor suppressors or oncogenes. Studies have shown that overexpression of miRNA family members such as miR-125 (Klusmann et al., 2010), miR-22 (Song Su et al., 2013), miR-155 (Eis et al., 2005), and miR-126 (Lechman Eric et al., 2012) can lead to malignant transformation. Mechanistically, miRNA’s crucial role in self-renewal provides opportunities for leukemic stem cells to acquire self-renewal properties. Gene expression and proteome profiles performed in leukemia stem cells overexpressing miR-126 led to the identification of the PI3K/AKT axis as targets of miR-126 important for chemotherapy resistance and quiescence (Lechman Eric et al., 2012). Studies have reported that miR-29 directly targets *DNMT3A* which in turn leads to the maintenance of self-renewal. In AML samples, miR-29 is upregulated in HSCs, and its overexpression in murine HSPCs led to the development of MPNs with progression to AML (Han et al., 2010). Although these studies suggest that miR-29a may have a role in initiating AML, contrasting studies have demonstrated that overexpression of miR-29a, leads to decreased AML cell proliferation and survival (Garzon et al., 2009). More recently, it was found that *DNMT3A*-mutant AML samples overexpress miR-196 family members, and morpholino knockdown of miR-196b in AML cells isolated from moribund FLT3ITD *Dnmt3a* heterozygous knockout mice induced significant cell death compared to negative control (Gamlen et al., 2022).

Similar to miRNAs, lncRNAs (∼200 nt) are also heterogenous and play vital roles in lineage specification by regulating gene expression by directly interacting with transcription factors, acting as a decoy for transcription factors, recruiting chromatin modifiers to target gene promoters, or by directly interacting with DNA to produce RNA-DNA hybrids (Nobili et al., 2016). In normal hematopoiesis, studies have shown that lncRNA-EC7 is required for the activation of BAND 3, HOTAIRM1 located between *HOXA1* and *HOXA2*, is essential for myeloid differentiation (Zhang et al., 2009), and functions as a miRNA sponge (Chen et al., 2017). In *NPM1*-mutated AML, HOTAIRM1 is highly expressed and contributes to leukemia cell autophagy and proliferation through increasing ULK3 expression by sponging miR-152–3p (Jing et al., 2021).

### 5.3 RNA modifications

Improvements in sequencing technologies have established a connection between RNA epigenetics and chromatin structure, divulging the roles of RNA modifiers and RNA binding proteins (RBPs) in gene regulation and genome function. Chromatin-associated RNAs can occur via *cis* interactions, where newly transcribed RNA remains at the site of synthesis, and t*rans* interactions, where RNA is released from their transcription sites to interact with DNA-binding proteins. Similar to epigenetic regulation, RNA modifications are carried out by enzymes that add methyl groups to RNA (writers), remove methyl groups from RNA (erasers), and proteins that recognize and bind to RNA (readers). Although RNA modifications were described over 50 years ago, recent studies have uncovered that RNA modifications and their binding partners are dysregulated in cancer, creating unique opportunities for new therapeutics. Over 100 different chemical marks can modify RNA, and N^6^-methyladenosine (m^6^A) is one of the most abundant modifications in mammalian cells. Some of the additional marks include 5-methylcytosine (m^5^C), Adenosine-to-inosine (A-I) RNA editing, and Cytosine-to-uracil RNA editing (Qing et al., 2021).

**FIGURE 3:**
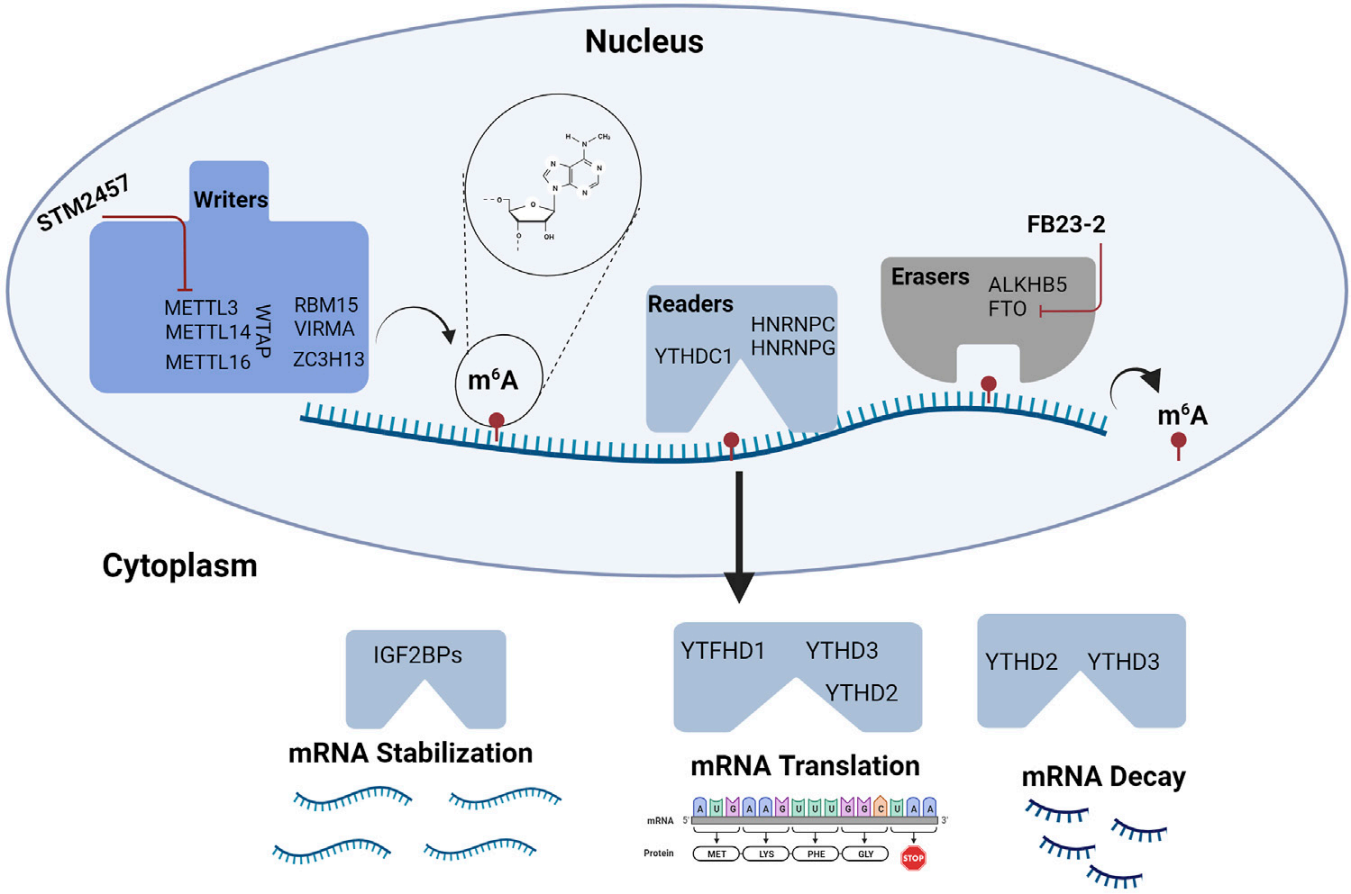
RNA Methylation in Hematopoiesis. RNA modifications and their binding proteins are emerging as key regulators of hematopoiesis. Modifications involving m6A are some of the most abundant modifications studied in HSPC biology. m6A is regulated by “writers” or RNA methyltransferase complexes and “erasers” or RNA demethylases. RNA function is modulated by m6A interactions with “readers” or specific proteins that affect mRNA stability, translation, and decay. Targeting m6A modifications may provide novel strategies for treating hematologic malignancies. To date, agents targeting METTL3 and FTO are being tested in leukemia. Figure made with BioRender.

Due to m^6^A being the most prevalent internal modification, many studies have focused on uncovering its role during normal and pathological states. The critical writers, erasers, and readers for m^6^A include the methyltransferase complex (METTL3-METTL14-WTAP), demethylases ALKBH5 and FTO, and YTH-domain family RBPs (YTHDF1-3, YTHDC1) (Figure 3) (Qing et al., 2021). Studies have demonstrated that m^6^A and its writer METTL3 are critical regulators implicated in the emergence of HSPCs during embryogenesis. Ablation of *Mettl3* in embryonic stem cells resulted in a significant decrease in m6A levels and impaired endothelial to hematopoietic transition. Deleting *Mettl3* in endothelial cells decreased the expression of *Runx1* and *Gfi1* and further impaired the development of HSPCs and downstream lineages (Lv et al., 2018). In adult hematopoiesis, *Mettl3* knockout reduced reconstitution of HSCs and blocked differentiation. However, ablation in cord blood led to cell differentiation and reduced cell proliferation, suggesting context dependent functions of Mettl3 (Vu et al., 2017). To establish a comprehensive landscape of m^6^A in the hematopoietic system, a new sequencing (SLIM-seq) strategy was developed for rare HSCs. The highest level of expression of m^6^A was observed in LT-HSCs when compared to downstream progenitors. Epitranscriptomic maps of HSPCs identified 8,599 m^6^A-tagged mRNAs, with about half coming from LT and ST-HSCs. Further profiling revealed that high m^6^A levels were present in LT-HSCs and uncovered that m^6^A -IGF2BP expression controls the transcriptional state and maintenance of HSCs (Yin et al., 2022). Mechanistically, IGF2BP was found to preserve mitochondrial homeostasis of HSCs by accelerating *Bmi1* mRNA decay. Other methyltransferases, such as METTL16, were shown to be vital in erythropoiesis by safeguarding genome integrity by controlling DNA response associated mRNAs (*Brac2* and *Fancm*). Mettl16 is expressed at high levels in erythroid progenitors, and deletion in erythroid cells leads to impairments in erythroid differentiation; therefore, altered expression may lead to hematological malignancies (Yoshinaga et al., 2022).

A genome-wide CRISPR-Cas9 knockout screen of 800 cancer cell lines revealed that *METTL16* was a strong dependency on AML cell lines. A small screen targeting DNA and RNA methylation genes demonstrated that *METT16* is essential for AML cell survival (Han et al., 2023). Homozygous conditional knockout of *Mettl16* in an *Mll-Af9* murine model inhibited leukemogenesis and prolonged the survival of primary AML models. Additionally, *Mettl16* was shown to have a significant role in leukemia stem cells and leukemia initiating self-renewal. Mechanistically, it was demonstrated that Mettl16 promotes the expression of BCAA metabolic proteins (Bcat1 and Bcat2) by adding m6A to their mRNA transcripts, further contributing to its oncogenic role in AML (Han et al., 2023).

*METTL3* is also associated with leukemogenesis and is upregulated in multiple AML cell lines (Vu et al., 2017). Deleting *METTL3* in human cell lines increases cell differentiation and induces cell cycle arrest (Barbieri et al., 2017; Vu et al., 2017). Consistently, *c-MYC* was identified as a transcriptional target of m6A-METTL3 oncogenesis either directly or through the transcription factors *SP1* and *SP2* (Barbieri et al., 2017; Vu et al., 2017)*.* Furthermore, it was also shown that METTL3 was recruited by binding protein CEBPZ to transcriptional start sites of active genes resulting in increased translation of *SP1.* Taken together, m6A modifications and their associated methyltransferases are important regulators with cell specific mechanisms that should be therapeutically explored.

## 6 Conclusion

Epigenetic regulation is a dynamic phenomenon that is critical in cell fate decisions during hematopoiesis. Understanding chromatin regulation through both histone modifications and methylation has identified how key regulators of hematopoiesis are silenced and activated during lineage specification. Disruption in any of these mechanisms leads to hematological malignancies of both myeloid and lymphoid lineages. In many instances these epigenetic regulators can function as either tumor suppressors or oncogenes depending on the disease and stage of development. The heterogeneity seen in leukemogenesis presents a critical challenge in targeting epigenetic enzymes and the associated patterns of genes dysregulated in hematological diseases. Targeting vital regulators in hematopoiesis with a combination of therapies represents a great avenue in treating aggressive hematologic malignancies, however, some studies have led to less than favorable outcomes (Issa et al., 2015; Liva et al., 2020). Therefore, it is important to further decipher the crosstalk mechanisms within the epigenome. For instance, evidence suggest that there is an interplay between DNA methylation and histone modifications however, mechanisms on how both cooperate to regulate differentiation needs further investigation. Advancements in new technology has not only expanded our knowledge of HSC decision making but it has also identified RNA modifications as an additional regulator of gene expression. RNA modifications such as m6A were shown to have regulatory effects on transcription, revealing a direct cross-talk mechanism between chromosome-associated regulatory RNAs and chromatin state (Liu et al., 2020). In addition, RNA methylation may play a role in immune surveillance. Although agents targeting cells with mutant RNA splicing factors have had mixed results in early clinical trials, the development of synthetic introns may provide more selectivity for mutant MDS or AML cells (North et al., 2022).

Although, we understand the established functions of epigenetic regulation in hematopoiesis, less is known about the non-canonical functions that may be exploited during disease states. A recent study deciphering mechanisms needed to switch between stem and differentiated states, found that an increase in splicing regulated by DNMT3A was needed to govern the exit (Ramabadran et al., 2023). The study reported that use of a spliceosome modulator was also shown to lead to sensitivity in isogeneic cell lines and decreased the leukemic burden *in vivo.* In many myeloid malignancies *DNMT3A* as well as *TET2* are commutated with splicing factor mutations. Thus, further molecular insights into how epigenetic regulators and splicing factors cooperate during malignancies will not only lead to advancements in selective inhibition but will outline additional roles DNA methylation has in stem cell biology.
